# Genomic regions in crop–wild hybrids of lettuce are affected differently in different environments: implications for crop breeding

**DOI:** 10.1111/j.1752-4571.2012.00240.x

**Published:** 2012-02-23

**Authors:** Yorike Hartman, Danny A P Hooftman, Brigitte Uwimana, Clemens C M van de Wiel, Marinus J M Smulders, Richard G F Visser, Peter H van Tienderen

**Affiliations:** 1 Institute for Biodiversity and Ecosystem Dynamics, Universiteit van AmsterdamAmsterdam, The Netherlands; 2 Centre for Ecology and HydrologyWallingford, UK; 3 Wageningen UR Plant BreedingWageningen, The Netherlands

**Keywords:** crop–wild hybrids, fitness, hitchhiking, *Lactuca*, mitigation strategy, quantitative trait loci, selection, transgenic plants

## Abstract

Many crops contain domestication genes that are generally considered to lower fitness of crop–wild hybrids in the wild environment. Transgenes placed in close linkage with such genes would be less likely to spread into a wild population. Therefore, for environmental risk assessment of GM crops, it is important to know whether genomic regions with such genes exist, and how they affect fitness. We performed quantitative trait loci (QTL) analyses on fitness(-related) traits in two different field environments employing recombinant inbred lines from a cross between cultivated *Lactuca sativa* and its wild relative *Lactuca serriola*. We identified a region on linkage group 5 where the crop allele consistently conferred a selective advantage (increasing fitness to 212% and 214%), whereas on linkage group 7, a region conferred a selective disadvantage (reducing fitness to 26% and 5%), mainly through delaying flowering. The probability for a putative transgene spreading would therefore depend strongly on the insertion location. Comparison of these field results with greenhouse data from a previous study using the same lines showed considerable differences in QTL patterns. This indicates that care should be taken when extrapolating experiments from the greenhouse, and that the impact of domestication genes has to be assessed under field conditions.

## Introduction

Hybridization between crop and wild relatives occurs for many crops in at least part of their geographic range. Molecular evidence for transfer of nontransgenic crop alleles to wild relatives has been found for a variety of crop species (Kwit et al. 2011). This includes crops that were beforehand thought to be of very low introgression risk, such as soybean and common bean (Stewart et al. 2003; Kwit et al. 2011), suggesting that hybridization between crops and their wild relatives is a more common phenomenon than previously considered (Ellstrand 2003). In addition, escape of transgenes for herbicide resistance from commercially grown crops into wild relatives is reported for at least 14 individual events in North America (Ellstrand in press), for example in oilseed rape (Warwick et al. 2008).

At present, there are no studies showing evidence for any potential negative ecological consequences of gene flow from transgenic crops to wild relatives (Kwit et al. 2011), such as increased invasiveness of the wild relative. Nevertheless, the approval of new transgenic crops is very stringent (EFSA 2011), and scientists and crop breeders are searching for methods to minimize the likelihood of transgene escape. Several model studies have addressed which factors are most important to the spread of crop alleles after a hybridization event. These studies suggest that hybrid fitness is one of the most important factors and that a selectively advantageous gene can spread rapidly in spite of very low gene flow pressure (Huxel 1999; Haygood et al. 2004). If so, the fitness of a transgene and natural selection acting upon it could be more important than rates of gene flow (Chapman and Burke 2006).

It has therefore been suggested that transgenes placed in close linkage with an allele that is selected against in the wild are more likely to be purged from the wild population (Gressel 1999; Stewart et al. 2003); we will refer to this mitigation strategy as a ‘purging strategy’. The basis for such a purging strategy is the fact that chances for introgression of transgenes into a wild relative depend, on the one hand, on gene flow and/or propagule pressure, but even more so, on the fitness of initial hybrids and the fitness effect of transgenes in the wild genomic background (Ellstrand 2003).

Consequently, the fate of a transgene does not only depend on the fitness effect of the transgene itself, but also on the genes around it. If a transgene is linked to a crop allele that is positively selected for in the wild habitat, genetic hitchhiking could cause the transgene to spread even if the transgene is selectively neutral or even mildly deleterious (Stewart et al. 2003). Alternatively, if a transgene is placed in close linkage with a gene or genomic block that causes a lower fitness in the wild habitat compared to the wild relative, it will have a smaller chance to introgress (Gressel 1999; Stewart et al. 2003). A purging mitigation strategy was already experimentally tested in tobacco (Al-Ahmad et al. 2004) and oilseed rape hybrids (Rose et al. 2009), where a transgene was placed in close linkage with a dwarfing gene. In both cases, there was a dramatic reduction in the survival of transgenic hybrid individuals carrying the dwarfing gene. This confirms that the location where a transgene is placed within the crop genome can be of vital importance to the probabilities of introgression.

Many studies on hybrid fitness are conducted in the greenhouse or solely in an agricultural setting as opposed to realistic field conditions for the wild species (Hails and Morley 2005). Conclusions based on these experiments might be misleading because Genotype × Environment (G × E) interactions can cause different selection pressures between a controlled greenhouse setting and variable field conditions (Weinig et al. 2002; Martin et al. 2006; Latta et al. 2007). For example, crop alleles might be favored in a greenhouse pot experiment, whereas in more competitive environments, wild alleles could be favored.

Moreover, there is an overall lack of information regarding genes or genomic blocks under selection in the field (Hails and Morley 2005). It would be valuable for risk assessment, as proposed by EFSA (2011), to know in which crop–wild systems, there are regions in the crop genome that are more or are less likely to introgress, to assess the effectiveness of a purging strategy. Quantitative Trait Loci (QTL) analysis allows pinpointing the location of regions under selection, and the traits associated with these regions. To our knowledge, only a few studies on crop–wild hybrids have used QTL analysis for this purpose (Baack et al. 2008; Dechaine et al. 2009).

We use the crop lettuce (*Lactuca sativa* L.), a leafy vegetable, and its wild relative prickly lettuce (*Lactuca serriola* L.) as a crop–wild model system. In the past 50–60 years, *L. serriola* has expanded its range dramatically in Western Europe (Hooftman et al. 2006; D’Andrea et al. 2009). In a series of field experiments, Hooftman et al. (2005, 2007, 2009) showed that at least four generations of lettuce crop–wild hybrids had higher germination and survival rates than the wild parent. Further genetic analysis showed that crop alleles were favorable at some loci, but disfavored at others, suggesting the possibility for genetic hitchhiking as well as purging (Hooftman et al. 2009, 2011). Lettuce might be a good candidate for transgene mitigation strategies, because it is a predominantly selfing species. This means the initial linkage disequilibrium (LD) in first-generation hybrids decays slowly, and selection will effectively act on large genomic blocks rather than on individual loci (Flint-Garcia et al. 2003).

In this study, we use recombinant inbred lines (RILs) from a cross between the cultivated Iceberg lettuce (*L. sativa* cv. Salinas) and *L. serriola* (UC96US23) (Johnson et al. 2000) to analyze the effects of selective field conditions on hybrid fitness and QTL analysis to identify genomic regions under selection. We identified QTL in field experiments for a broad set of fitness and fitness-related traits at different life stages relevant to the success of *Lactuca* hybrids in the field. In addition, we compare these field results with domestication-related QTL from the same RIL population grown in the greenhouse (Y. Hartman, D.A.P. Hooftman, M.E. Schranz and P.H. van Tienderen, unpublished data).

Because the genomic location of crop (trans)genes can be of vital importance for the chance and rate of introgression, we studied the selection on genomic regions in different environments. Specifically, we addressed the following questions: (i) Which traits are important for fitness in the field and do crop alleles confer a selective (dis-)advantage? (ii) Are there regions where crop alleles provide such negative fitness effects that they could be effective in a purging strategy? (iii) How important is G × E? In particular, how do field QTL compare to greenhouse QTL and can small-scale contained greenhouse experiments be used to assess potential ecological consequences? The results are a first step in establishing whether the genomic location of a transgene in the crop is important for predicting its fate if outcrossing occurs to wild relatives.

## Material and methods

### Plant material

We used an existing RIL population from a cross between a crop species lettuce (*L. sativa* cv. Salinas) and its wild relative species Prickly lettuce, originally collected in California, USA (*L. serriola* UC96US23; Johnson et al. 2000; Argyris et al. 2005; Zhang et al. 2007). These two fully interfertile species (Koopman et al. 2001) show marked differences in phenotype. *Lactuca serriola* has long serrate leaves that contain white bitter latex. Plants have *ca.* 2-mm-long spines on the stem base and on downside leaf midribs. The wild-type produces almost no head, instead it bolts and flowers early and can develop many basal and cauline reproductive shoots. In contrast, *L. sativa* cv. Salinas typically has broad almost circular leaves, without any spines or latex content. It develops a very dense head without any basal side shoots and bolting is delayed (de Vries 1997).

*Lactuca serriola* mainly occurs in ruderal habitats, such as roadsides, railways, and construction sites. It is an annual species that flowers in July–August and survives winter as seed, but sometimes as small rosettes (Y. Hartman, personal observation). Lettuce is a predominantly selfing species, but up to 5% outcrossing rates from crop to wild relatives via insect pollination have been reported (D’Andrea et al. 2008; Giannino et al. 2008).

### Field design and traits measured

We selected two tilled field sites with contrasting environments. The first site, Sijbekarspel (SB), the Netherlands (N52°42′, E04°58′), has a clay soil mimicking agricultural conditions with nutrient rich and high water retention conditions. Wageningen (WG), the Netherlands (N51°59′, E05°39′), has a nutrient poor, dry, sandy soil, more similar to the natural habitat of *L. serriola*. In SB, environmental data were obtained with a data logger, measuring temperature and humidity levels. In WG, daily temperature and rainfall was obtained from the Haarweg weather station approximately 1 km from the field (http://www.maq.wur.nl/UK/).

Ninety-eight RILs and their parent lines were sown on April 27–29, 2010, in SB and 1 week later, on May 3, 2010, in WG. The experiment lasted until the end of October to be able to follow the entire life cycle. Each site was subdivided into 12 blocks, each block containing all RILs and the parental lines. Each block was subdivided into 200 squares of 40 by 40 cm, laid out in five rows of 40 squares, and spaced 10 cm from each other; blocks were spaced 80 cm from each other. Within each block, the 100 lines were randomly assigned to squares. In each square, we initially sowed 30 seeds. The remaining 100 squares per block were used for another associated study (Uwimana 2011).

During the life cycle, we measured several fitness-related traits (Table 1). Germination and initial establishment were measured by counting the number of seedlings 4 weeks after sowing. At this stage, we first hand-weeded both sites, because seedlings were fully overgrown, and then thinned the number of lettuce seedlings to five per square. The seedlings were selected based on their position in the square to achieve uniform spacing. We collected two individuals per square for biomass measurements 7 weeks after sowing. Biomass samples were dried for 3 days at 70°C. One week later, we did a last thinning round so that one individual (the one closest to the center of the square) was left per square for measurements in the adult stage. We recorded the flowering date, and at seed set, we counted the number of basal reproductive side shoots, the number of branches of the main stem, and the total number of seeds in 10 capitula to calculate the average number of seeds per capitulum. Subsequently, we estimated the total number of capitula from the number of branches and shoots following Hooftman et al. (2005, see Data S1), and the seed output of a reproductive plant as the product of the number of capitula and the average number of seeds per capitulum. Survival rate was calculated as the proportion of seed-producing plants per line using the 12 data points per RIL (one individual per square). Finally, seeds produced per seed sown (SPSS) was used as ‘main fitness trait’, because it is the closest direct association with lifetime fitness of the different lines, and calculated as:


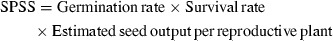
(1)

**Table 1:** Traits examined in a *Lactuca sativa* cv. Salinas × *Lactuca serriola* recombinant inbred lines (RIL) population

Plant stages	Traits	Abbreviation	Evaluation method
Seedling	Germination rate	GM	No. of seedlings 4 weeks after sowing divided by the total amount of seeds sown, values arcsine-square-root-transformed
Rosette	Biomass (g)	BM	Dry weight of two rosettes divided by two, values log-transformed
Flowering	Days to first flower (day)	FLD	No. of days from sowing to flowering of first flower, values log-transformed
Seed set	No. of reproductive basal shoots (count)	SHN	No. of basal side shoots which have flower buds, flowers and/or seed head, values log-transformed
	No. of branches main inflorescence (count)	BRN	No. of branches counted from the base of the main inflorescence to the top, values log-transformed
	No. of seeds per capitulum	SDC	Average no. of seeds per capitulum based on 10 collected capitula
	Total no. capitula	TC	Total no. of capitula developed, calculation following Hooftman et al. (2005); values log-transformed
	Seed output	SDO	Total no. of seeds produced, calculation following Hooftman et al. (2005); values square-root-transformed
	Survival rate	SUR	No. of plants per RIL that produced seed divided by 12, values arcsine-square-root-transformed
	Seeds produced per seed sown	SPSS	No. of seeds per seed sown, calculated by multiplying germination rate, with survival rate and seed output, values square-root-transformed

The QTL found in this study are compared, in genomic location, to QTL that are based on data obtained in a separate study conducted under uniform greenhouse conditions in 2009 using an extended RIL set (114 RILs, one individual per RIL) and analyzed using the same genetic map (Y. Hartman, D.A.P. Hooftman, M.E. Schranz and P.H. van Tienderen, unpublished data).

### Statistical analysis

All statistical analyses were performed in PASW Statistics 17.0 (SPSS Inc. 2009). We estimated the mean, standard deviation, and selection differential for each trait separately. Selection differentials were calculated as the covariance between the main fitness trait, SPSS, and the separate trait values, using the 12 data points per RIL (one per square) as replicates. The composite traits, number of capitula, seed output, and SPSS were generated using the original nontransformed data to maintain the link with fitness. Differences in variance distribution among the individual traits forming composite traits could lower the power to detect QTL. However, statistical tools for QTL analyses of composite measures are still lacking. Prior to the estimation of heritability values and QTL analyses, all traits were transformed. This improved normality of the distributions, with the exception of number of seeds per capitulum as it was already normally distributed. Germination and survival rates were expressed as proportional data and arcsine-square-root-transformed. Biomass, number of reproductive basal shoots, number of branches, and total number of capitula were log-transformed. Seed output and SPSS were square-root-transformed. Broad-sense heritability was estimated as the proportion of the total variance accounted for by the genetic variance (Visscher et al. 2008).

### Quantitative trait loci analysis

Genetic map and marker data used in the QTL analysis were obtained from The Compositae Genome Project website, which is supported by the USDA IFAFS program and NSF Plant Genome Program. The genetic map employed consisted of 1513 markers distributed over nine linkage groups (http://cgpdb.ucdavis.edu/GeneticMapViewer/display/; map version: RIL_MAR_2007_ratio; Johnson et al. 2000; Argyris et al. 2005; Zhang et al. 2007). All QTL analyses were performed with Composite Interval Mapping (CIM) in QTL Cartographer version 2.5.008 (Wang et al. 2010). Tests for the presence of a QTL were performed at 2 cM intervals using a 10 cM window and five background cofactors that were selected via a forward and backward stepwise regression method. Statistical significance threshold values (α = 0.05) for declaring the presence of a QTL were estimated from a 1000 permutations (Churchill and Doerge 1994; Doerge and Churchill 1996). One-LOD support intervals and additive effects were calculated from the CIM results. QTL analyses were performed on the data of both locations separately. The linkage map and QTL were drawn with MapChart 2.2 (Voorrips 2002). The two field sites were analyzed separately, after which sites and greenhouse QTL were compared. Hence, overlap in QTL location is identified in case a QTL is significant among the separate environments. This differs from the mixed model approach of Mathews et al. (2008) in which QTL main effects and QTL by environment interactions are estimated jointly.

### Effect sizes of fitness quantitative trait loci

To quantify and depict the strength of selection pressure on fitness QTL, we estimated the average SPSS for genomic locations where fitness QTL clustered for both field locations. We estimated the effect size in SPSS for containing either crop or wild alleles at these locations, and for the combinations thereof. This was performed for both sites separately. We included 73 RILs for which we could unambiguously determine the genotype for those specific genomic locations, i.e., no missing data or all present loci of one parental background.

## Results

### Environmental data

The summer of 2010 was relatively warm and in August also relatively wet. Weather conditions were comparable in SB and WG. From May until the end of October, the average temperatures were 15.5 and 14.8°C and relative humidity was 85.2% and 79.5%, respectively. In SB, the maximum temperature reached 39.9°C in July and a minimum of −1.8°C in October; in WG, the maximum temperature reached 39.5°C in July and a minimum of −3.6°C in May. The total number of plants, based on one plant per square, that survived until reproduction was also very similar with 56.9% of plants surviving in SB and 57.1% in WG.

### Broad-sense heritability and selection differentials

Broad-sense heritability values ranged from 14.1% to 89.5% (Table 2). Germination rate, biomass, and branch number showed much lower heritability than the other traits. The highest heritability was found for days to first flower. All traits had significant selection differentials and all selection differentials favored higher values for all traits, except for days to first flower where up to 7–8 days earlier flowering was favored.

**Table 2:** The mean within line standard deviation, broad-sense heritability values, and selection differentials for the parent lines and the recombinant inbred lines (RIL) population

	Crop		Wild		RILs			Selection differential	
					
Traits	Mean	SD	Mean	SD	Mean	SD	Heritability (%)	Absolute	Standardized
Field fitness Sijbekarspel									
GM (%)	60.8	18.9	25.3	11.0	52.8	14.6	27.9	4.6	0.314**
BM (g)	1.083	0.586	0.542	0.334	0.681	0.335	14.1	0.037	0.111*
FLD (day)	115.0	–	93.8	3.1	94.3	4.8	89.5	−8.0	−1.677**
SHN	–	–	4.2	2.1	3.3	1.9	50.5	1.2	0.609**
BRN	–	–	36.3	8.0	28.3	5.2	14.1	4.1	0.778**
SDC	–	–	10.0	3.4	7.2	2.4	75.8	7.0	2.876**
TC	–	–	2566	492	2011	421	62.0	421	0.999**
SDO	–	–	26 034	11 445	14 411	6179	73.6	18 751	3.035**
SUR (%)	0.0	–	100.0	0.0	56.9	14.6	76.4	43.1	2.952**
SPSS	0	–	6921	4757	4450	2464	78.4		
Field fitness Wageningen									
GM (%)	72.8	21.2	35.0	14.1	66.4	18.0	24.4	7.7	0.427**
BM (g)	1.543	0.818	0.955	0.593	1.183	0.554	16.5	0.061	0.110*
FLD (day)	104.0	–	82.1	3.3	91.4	4.5	89.5	−7.4	−1.652**
SHN	–	–	1.3	0.9	2.4	1.3	54.6	0.9	0.728**
BRN	–	–	41.8	8.2	29.1	5.5	16.5	4.4	0.811**
SDC	–	–	19.1	3.1	12.6	2.6	64.0	3.7	1.388**
TC	–	–	2333	433	1887	351	74.9	390	1.110**
SDO	–	–	45 171	11 869	23 631	6865	68.1	13 790	2.009**
SUR (%)	0.0	–	100.0	0.0	57.1	12.9	80.0	42.9	3.319**
SPSS	0	–	15 743	7638	8464	4129	80.2		

For abbreviations, we refer to Table 1. *Significant at 0.05 level, **Significant at 0.01 level.

### Quantitative trait loci analysis

In this study, we detected a total of 49 QTL for 10 fitness and fitness-related traits (Table 3), although the actual number of unique QTL could be lower because of the measurement of several hierarchically related fitness traits. The range of Phenotypic Variation Explained (PVE) per QTL varied between 7.2% and 48.0%. QTL were distributed over eight linkage groups; no QTL were found on LG1. For each trait, one to five QTL were detected (mean 2.5). The 1-LOD support intervals ranged from 0.6 to 13.3 cM (mean = 3.3 cM).

**Table 3:** Quantitative trait loci (QTL) positions using composite interval mapping in a *Lactuca sativa* cv. Salinas × *Lactuca serriola* recombinant inbred lines population

LG	Traits	Position	1-LOD interval	Effect	PVE	LOD	Position	1-LOD interval	Effect	PVE	LOD	GH
Sijbekarspel							Wageningen					
1	nd											
2	SUR	101.1	98.9–102.3	−0.19	8.3	3.8						
	SUR						106.9	106.4–108.4	−0.21	9.1	4.5	
	FLD						106.9	106.3–107.1	0.03	13.0	5.9	Y
	SDC						121.6	120.8–123.9	1.53	12.9	3.6	
3	SDO	41.0	38.6–41.6	−30.78	25.3	7.7	41.6	40.2–42.7	−18.29	20.5	7.0	Y
	SHN	44.9	42.4–46.2	−0.10	10.2	4.6						Y
	TC	44.9	44.2–48.2	−0.05	14.3	5.1						L
	SHN	66.8	66.1–69.1	−0.10	9.6	4.4						L
4	SUR						112.8	111.4–114.8	−0.18	7.2	3.6	
	GM						125.2	124.4–126.3	−0.06	18.4	6.2	
	BRN	162.4	160.9–162.8	−0.05	19.0	5.8						
5	BRN	31.4	30.0–31.7	−0.04	13.1	4.4						
	BRN						125.1	121.9–127.0	0.05	13.3	4.0	
	TC						125.1	122.5–126.8	0.05	10.0	3.6	
	SDC	148.0	146.9–151.9	2.06	15.3	3.9	148.0	146.8–151.9	1.63	13.6	3.5	
	SPSS	148.0	147.2–150.2	14.17	9.5	3.8	148.0	146.9–150.7	19.33	9.9	4.3	NA
	SDO	148.0	147.3–149.3	32.07	29.4	8.5	148.0	147.4–151.1	20.75	28.8	8.8	
6	BM	15.5	14.3–17.9	−0.02	12.5	4.7						NA
	BM						29.1	28.4–30.3	−0.02	13.3	4.8	NA
	BM						35.9	35.4–37.7	−0.02	14.0	5.1	NA
	BM						58.8	56.2–59.7	0.02	11.1	3.9	NA
7	BM	15.3	14.0–16.4	0.02	15.1	6.1						NA
	SHN	15.2	14.4–15.5	0.18	27.6	10.3	19.9	19.0–22.2	0.19	37.8	11.7	Y
	TC	15.3	13.7–18.5	0.07	19.8	6.3	15.5	14.5–18.5	0.06	14.9	5.0	
	FLD	18.4	17.4–18.5	0.05	42.9	14.6	19.9	19.2–22.1	0.05	48.0	15.9	Y
	SUR	18.5	18.2–18.9	−0.40	34.5	13.4	19.9	19.5–22.2	−0.42	36.6	13.0	L
	SPSS	18.5	18.4–20.9	−20.13	19.5	7.8	19.9	18.5–29.3	−27.78	20.5	8.2	NA
	BRN	75.1	72.6–75.9	−0.04	13.3	4.1						L
	SPSS	76.7	75.1–77.1	−29.93	16.2	6.6						NA
8	SHN	23.4	22.1–25.4	−0.09	8.8	3.9	22.1	20.7–23.4	−0.10	12.2	4.7	
	TC	23.4	22.1–25.7	−0.05	10.6	3.8						
	BRN						60.3	59.2–61.2	−0.06	16.1	4.9	
	GM	113.4	113.0–117.4	0.04	10.2	3.6						
	BM	119.0	117.7–120.1	0.01	10.5	4.5						NA
9	BM						60.6	60.4–61.0	0.02	16.7	6.0	NA
	BM	72.3	71.2–84.5	0.02	17.3	6.8	70.3	69.4–71.3	0.02	17.9	6.3	NA
	GM	70.3	69.4–74.4	0.04	12.2	4.2						
	GM						82.6	81.7–85.4	0.05	11.2	4.1	

For abbreviations, we refer to Table 1. Positive additive effects indicate that the crop-type (*L. sativa*) allele increases trait values, whereas negative values indicate that the wild-type (*L. serriola*) allele increases trait values. PVE, Percentage of variation explained; nd, no QTL detected. QTL with peak values within 5 cM are shown on the same line. GH indicates overlap with greenhouse results (Y. Hartman, D.A.P. Hooftman, M.E. Schranz and P.H. van Tienderen, unpublished data), with Y indicating peak values within 5 cM, L indicating same linkage group but different position, NA indicating a trait was not measured in the greenhouse, and finally blanks indicate that no QTL was found on that linkage group.

**Figure 1:**
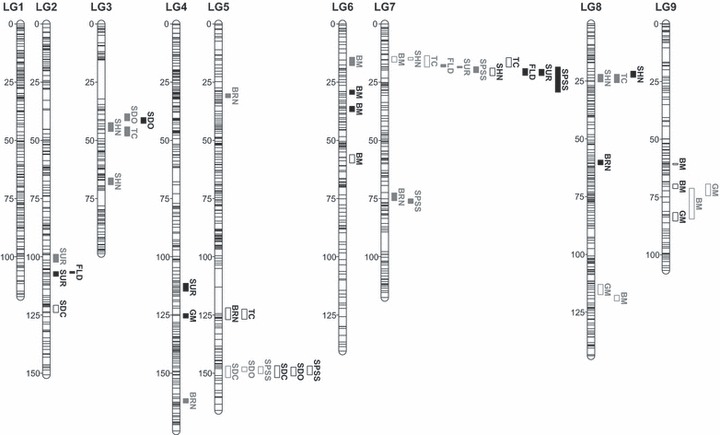
Genomic locations of quantitative trait loci (QTL) detected in composite interval mapping. The map consisted of 1513 markers indicated by horizontal lines on the linkage group bars, and map distances (cM) are shown on the left side. Bars to the right represent one LOD confidence intervals of QTL. For abbreviations, we refer to Table 1. An open bar indicates that the crop-type (*Lactuca sativa* cv. Salinas) gives a selective advantage, whereas a filled bar indicates that the wild-type (*Lactuca serriola*) gives a selective advantage. Selective advantage is inferred from the selection differentials (Table 2). Bar colors indicate the location: Gray = Sijbekarspel (SB) and Black = Wageningen (WG).

For almost every trait, we found more than one QTL for both field sites. The majority of the traits, including seeds produced per seed sown, showed opposing allele effects. This means that for the same trait, values were increased by the crop allele at some loci, whereas at other loci, the increase came from the wild allele (Table 3). When field QTL for the same trait co-localized, additive effects were always in the same direction. Crop alleles invariably increased trait values for days to first flower and seeds per capitulum. In the case of days until first flower, the crop allele caused a negative fitness effect (following the selection differentials) with a delay in flowering, but in the case of seeds per capitulum, this caused a positive fitness effect with a higher amount of seeds per capitulum. The crop allele conferred a selective advantage for 45% of the QTL found (Fig. 1). In contrast, the wild allele invariably conferred the selective advantage for survival rate.

There were considerable differences between the two field sites combined and previous greenhouse QTL results (Table 3, Y. Hartman, D.A.P. Hooftman, M.E. Schranz and P.H. van Tienderen, unpublished data). There were eight fitness-related traits that were previously mapped in the greenhouse (Table 3). Biomass was not measured in the greenhouse. In addition, germination was not measured as percentage of germinated individuals, but as germination speed (time when 50% of seeds had germinated). Therefore, we did not determine the overall main fitness for the greenhouse. Combining the field results, we found 27 QTL present at either one or both sites for these eight traits (Table 3, Fig. 1). Only five of these overlapped with greenhouse QTL on the same linkage group, including two for days to first flower, two for shoot number and one for seed output. An additional four QTL occurred on the same linkage group but at a different location, including survival rate. The other 18 QTL were unique to the field.

Combining the two field sites for all 10 traits, we found 38 distinct QTL, of which 11 QTL were found at both sites. The majority of traits showed at least one QTL that co-localized for both sites, except for germination rate. However, only two regions showed a clustering of several QTL that included the main fitness QTL, namely at the bottom of LG5 and at the top of LG7 (Fig. 1). The crop allele conferred the selective advantage for all QTL at LG5, increasing seeds per capitulum, seed output, and seeds produced per seed sown. At LG7, both the cultivar and the wild allele were favored for different traits; the crop allele increased biomass, shoot number, and total capitula, whereas the wild allele reduced days to first flower and increased survival rate and seeds produced per seed sown.

### Effect sizes of fitness quantitative trait loci

The fitness strength, as indicated by the average seeds per seed sown, differed considerably between crop and wild alleles at the two genomic locations, LG5 and LG7, where fitness QTL were found. Within 73 RILs used, 21 RILs had the crop genotype at LG5 and LG7, 23 RILs had the wild genotype at both locations, 16 RILs had the crop genotype at LG5 and the wild genotype at LG7, and 13 RILs had the opposite combination.

As described earlier, these two regions had opposing effects, which is confirmed by the effect sizes. The highest amount of seeds produced per seed sown was provided by a combination of crop alleles at LG5 and wild alleles at LG7 (8105 seeds in SB and 14 580 seeds in WG; Table 4), whereas the lowest amount was provided by the opposite combination (193 seeds in SB and 1767 seeds in WG). To illustrate the individual location effects starting from a complete wild genotype, a change from wild to crop allele at LG5 meant an increase of 4326 (114%) seeds in SB and 7727 (112%) seeds in WG (Table 4). In contrast, at LG7 a change in a wild to crop allele meant a decrease of 3586 (94.9%) and 5086 (74.2%) seeds respectively.

**Table 4:** Effect sizes of the overall fitness quantitative trait loci expressed in seed output per seed sown for LG5 and LG7, for both sites separately

	Crop allele at LG7	Wild allele at LG7
Sijbekarspel		
Crop allele at LG5	4444	8105
Wild allele at LG5	193	3779
Wageningen		
Crop allele at LG5	8182	14 580
Wild allele at LG5	1767	6853

## Discussion

### Two main genomic regions are under selection

In this study, we clearly identified crop genomic regions with opposing selective directions, as indicated by the QTL for seeds produced per seed sown (SPSS), the main fitness trait. It involved two main genomic regions, one where the crop alleles were selectively beneficial (at the bottom of LG5) and one where crop alleles were negative for fitness (at the top of LG7). QTL and effect sizes results were consistent at both field sites; a change in genotype at these respective genomic locations coincided with quite large differences in SPSS. For a successful purging strategy where a transgene is placed in close linkage with a gene or genomic block that causes a lower fitness in the wild habitat (Gressel 1999; Stewart et al. 2003), clear indications are needed of those negatively selected regions. This should preferably involve several co-localized, fitness-related QTL, where the crop allele is selected against under various field conditions. For risk assessment, the opposite information is very valuable as well because it would indicate regions where the crop allele is selected positively and should therefore be avoided as places for transgene insertion.

At LG5, the fitness QTL co-localize with two traits for which the crop allele consistently conferred a selective advantage favoring a higher seed output and more seeds per capitulum. In contrast, at LG7, the wild allele conferred selective advantages by favoring earlier flowering and a higher survival rate, whereas for other traits in that region, the crop rather than the wild allele conferred the selective advantage. A QTL analysis does not allow conclusions on whether the clustering of QTL is because of the pleiotropic effects of a major gene or because of close linkage of several genes (Erickson et al. 2004). At LG7, it seems most plausible to be pleiotropy: delayed flowering, induced by the crop allele, might be correlated to a higher total capitula and shoot number, but with fewer seeds per capitulum and a lower survival (see Table S1). The latter ultimately leads to a lower fitness for hybrids with this crop genomic block. This suggests that a transgene inserted in LG7 is much less likely to introgress into the wild population through crop–wild hybrids than a transgene in LG5 (Stewart et al. 2003).

Similar patterns of few fitness QTL with contrasting selective directions have been shown for sunflower (Baack et al. 2008) and slender wild oat (Latta et al. 2010), suggesting that it is not uncommon that there are crop genomic blocks that are negatively as well as blocks that are positively selected for in the wild habitat. On the basis of marker comparisons, previous lettuce research with hybrids and backcrosses of a different cultivar and *L. serriola* from the Netherlands and consequently a different linkage map shows that only the region at the bottom of LG5 concurs as a region where crop alleles are favored (Hooftman et al. 2011). There were no similarities between regions where the crop alleles were selected against. This shows that results cannot be extrapolated readily across different cultivars, but rather should be viewed case-by-case as is carried out for all new events under the current risk assessment (EFSA 2011).

### Selection pressures on traits and cultivar alleles

Selection pressures were similar between our two field locations as indicated by the selection differentials. The weather data indicated that conditions were very similar between the sites, no apparent differences were observed in herbivore or pathogen damage, so that soil type (clay versus sandy soil) presumably was a main selective difference between the two sites.

At both sites, higher values were favored for all traits except for days to first flower where early flowering was favored up to 7–8 days. Many ruderal annual species, such as *L. serriola*, exhibit fast development and early flowering especially under stressful conditions (Mercer et al. 2007). Delayed flowering may therefore have been a target of selection during domestication of leafy vegetables, explaining the genetic variation in flowering time and selection for earliness in the segregating RIL population. The highest selection differentials were shown for seeds per capitulum, total capitula, seed output, and survival rates. We could not deduce exactly which of these underlying traits is most important for the main fitness trait, because traits were highly correlated with one another (see Table S1). Seed output and survival seem to play a more important role in selection than germination and biomass.

Paying specific attention to crop alleles, we found that the crop allele conferred the selective advantage for almost half of the QTL found (45%). For almost all traits, including the main fitness trait, more than one QTL was found and both crop and wild alleles conferred the selective advantage at different genomic locations. The actual number of QTL could be lower because of the fact that total capitula, seed output, and seeds per seed sown are composite traits based on other measured traits such as number of branches and seeds per capitulum, thus causing overlapping QTL in some instances. Nevertheless, in several instances, the crop allele confers the selective advantage. Moreover, our results concur with earlier work on lettuce with hybrids and backcrosses derived from other parental lines (Hooftman et al. 2011), where after two generations of selective sorting, allele frequencies where skewed in the direction of the crop allele for various loci, suggesting that crop alleles can indeed confer selective advantage in specific genomic regions. This mixed selective pattern of both crop and wild alleles has also been observed in sunflower (Mercer et al. 2007; Baack et al. 2008) and radish (Snow et al. 2010). This contrasts to the general assumption that typically crop traits do not spread readily into the wild because domestication genes confer some selective disadvantage to the hybrid individuals (Stewart et al. 2003; Hails and Morley 2005). Therefore, in lettuce as well as in other species, the introgression of crop alleles into their wild relatives might pose an ecological risk, although the genes presumably originate from a wild relative that was used in the breeding program of the crop. In that sense, it could differ from the potential risk of transgenes that are derived from unrelated (micro-) organisms (Dale 1999).

### Genotype × Environment interactions: greenhouse versus field

Most research on transgenes is conducted in controlled greenhouse environments or under agricultural field conditions (Hails and Morley 2005), testing for the effectiveness of the transgene (for its agronomic objectives, e.g., disease resistance) and to predict the potential effects of transgene escape. However, we show considerable differences between field and greenhouse QTL patterns; moreover, the region on LG5 where crop alleles were positively selected in the field was previously not identified in the greenhouse. Similar major differences have been found in other studies when QTL patterns between greenhouse and field were compared (Weinig et al. 2002; Malmberg et al. 2005; Martin et al. 2006; Gardner and Latta 2008). Therefore, extrapolation of greenhouse results into predictions for field situations, after gene escape, should be performed with great care (Mauricio 2001).

We see two main reasons for the large difference between greenhouse and field QTL results. First, heritability values are lower in the field because of an increase in environmental variance (Latta et al. 2007; Gardner and Latta 2008); this could affect the threshold at which QTL are statistically detectable, although sample sizes used in the field are often higher to compensate for this. Second, selection pressures might differ between greenhouse and field for different traits and loci (Weinig et al. 2002; Martin et al. 2006). For example, for slender wild oat, it was found that fitness in the greenhouse was mainly explained by days to first flowering, whereas fitness in the field was mainly explained by growth rate and size effects (Gardner and Latta 2006; Latta et al. 2007). Similarly, in Iris hybrids, alleles from the flood-tolerant parent line were favored in the field, whereas the flood-intolerant parent line was favored in the relatively dry greenhouse (Martin et al. 2006).

Genotype × Environment interactions can thus cause changes in selection pressures and subsequently which alleles are selected for (Mercer et al. 2007). Through mortality, hybrid variability interacts with the environmental and seasonal variation in the field (Weinig et al. 2003; Hails and Morley 2005), causing lineage sorting and selection not present in a greenhouse (Campbell et al. 2009; Hooftman et al. 2009). Therefore, for an environmental risk assessment, as discussed later, it is important to take variability in selective pressures into account, and use realistic field situations and not agricultural fields only (Hails and Morley 2005; Mercer et al. 2007). It is clear that G × E greatly complicates generalizations and requires extensive field trials, although it is not needed to do such experiments for each transgene event, it would be useful to know for which crop–wild systems position effects are relevant.

### Implications for GM crop risk assessment

Our results show that at different genomic locations, alleles are favored in opposite directions: at some locations crop alleles are selected against and at other locations they are selected for. In theory, these results could be used for designing a purging strategy that would depend heavily on identifying major QTL – as we identified here – that are uniformly selected toward one of the parental species. A few empirical studies showed that the speed of introgression indeed differs for different crop genomic regions (Snow et al. 2010; Hooftman et al. 2011). Snow et al. (2010) showed that some crop alleles and/or regions introgress easily, while other crop alleles remain rare or do not introgress at all. The result will be a mosaic with, on the one hand, major QTL that are either selected up- or downward in all environments and are less subject to G × E interactions. On the other hand, minor or intermediate QTL that may react differently in different environments (Morjan and Rieseberg 2004).

A purging mitigation strategy might be effective at inhibiting transgene escape if the following conditions are met. First, the transgene should be linked to a major QTL that is invariably selected against in the wild habitat, for example, the transgene might be linked to a crop genomic region with an extreme detrimental effect in the wild habitat but not in the agricultural situation (Chapman and Burke 2006). This should be verified in field trials across different environments (Hails and Morley 2005). For lettuce, prevention of bolting has been suggested previously as a possible major deleterious trait (Gressel 1999; Rose et al. 2009). In our data, we can link the major QTL region LG7 to a delay in flowering because all crop parent individuals died in the field trial being incapable to bolt. Second, none of the biotic mitigation strategies will be entirely fail-safe (Haygood et al. 2004). Therefore, a purging strategy should be combined with other mitigation strategies (Haygood et al. 2004; Lee and Natesan 2006; Kwit et al. 2011), further delaying transgene escape.

However, even tight linkage of a transgene to a negative block might not always prevent transgene escape when opportunity for hybridization is high (Linder et al. 1998). As time passes, LD breaks up because of recombination events and thus separating the transgene from the negatively selected block (Lee and Natesan 2006). However, before LD is broken, a strong directional selection could already have led to the rapid purging of deleterious genomic blocks via lineage sorting, just as it can lead to the rapid spread of favorable alleles (Rieseberg et al. 2002; Morjan and Rieseberg 2004) during selective sweeps. Under stressful conditions, this selection can happen in a few generations (Campbell et al. 2009; Fakheran et al. 2010; Hooftman et al. 2011).

Most crops are grown in rotations, so that repeated outcrossing events into already established hybrid populations could create several types of backcrossed hybrids. Heterosis effects in different artificially created hybrids have already been identified in Lettuce (Hooftman et al. 2005, 2007). It is difficult to predict whether purging of crop (trans-)genes will occur, depending on the interplay between the detrimental fitness effect of genomic regions, the continuous creation of new hybrids, and the breakdown of LD. Such scenarios are difficult to test experimentally but are currently under investigation using modeling approaches.

In the last decade, more and more genetic resources, such as genetic maps and markers, are becoming available, making it easier to study the effects of domestication genes in a wild genetic background for an increasing amount of crop–wild complexes (Collard and Mackill 2008). Possible methodological caveats could be the Beavis effect (Beavis 1998) where through interacting small sample sizes and low heritability values major QTL are quickly overestimated, leaving smaller QTL undetected. Another issue is that QTL results can vary considerably depending on which parental lines are used (Mauricio 2001; Collard and Mackill 2008). We will explore this further through a comparison made between these results and QTL patterns from another lettuce crop–wild cross. In addition, we will use these empirical field data in mathematical models simulating different scenarios regarding transgene escape chances, including multiple events.

### Data archiving

Data for this study are made available as electronic Supporting information.

## Supporting Information

**Data S1.** Data for this study are made available as electronic Supporting information.

**Table S1.** Pearson correlation coefficients.
